# Leukocyte-Rich Platelet-Rich Plasma Injections Do Not Up-Modulate Intra-Articular Pro-Inflammatory Cytokines in the Osteoarthritic Knee

**DOI:** 10.1371/journal.pone.0156137

**Published:** 2016-06-03

**Authors:** Erminia Mariani, Valentina Canella, Luca Cattini, Elizaveta Kon, Maurilio Marcacci, Berardo Di Matteo, Lia Pulsatelli, Giuseppe Filardo

**Affiliations:** 1 Laboratory of Immunorheumatology and Tissue Regeneration/RAMSES, Rizzoli Orthopaedic Institute, Bologna, Italy; 2 Department of Medical and Surgical Sciences, University of Bologna, Bologna, Italy; 3 Laboratory of Biomechanics and Technology Innovation/NABI, 2^nd^ Orthopaedic and Traumatologic Clinic, Rizzoli Orthopaedic Institute, Bologna, Italy; Magna Graecia University, ITALY

## Abstract

**Introduction:**

The presence of leukocytes in platelet concentrates is deemed to cause deleterious effects when injected intra articularly. The aim of this study is to analyse both local and systemic effects induced by leukocyte-rich Platelet-rich Plasma (PRP) injections through a proteomic characterization of serial synovial fluid and blood samples obtained from subjects treated for knee OA. Secondary aim was to compare the effects on knee homeostasis and systemic response with those obtained with visco-supplementation.

**Methods:**

Thirty-six OA patients treated either by autologous L-PRP or HA intra-articular knee injections, administered in series of three at one-week intervals, were analyzed. Just before the injection, 1 ml of synovial fluid was collected through the same needle way. In the same time, a peripheral blood sample was obtained and plasma separated. A further peripheral blood sample was collected at 2, 6, and 12 months. L-PRP, plasma and synovial fluid were tested by multiplex bead-based sandwich immunoassay by means of the Bio-Plex suspension array system (Bio-Rad Laboratories) for the presence of pro- and anti-inflammatory cytokines (IL-1beta, IL-6, IL-8, IL-17 and IL-4, IL-10, IL-13) and growth factors (FGF-b, HGF, PDGF-AB/BB).

**Results:**

In general, pro-inflammatory cytokine levels were similar at basal condition and after treatment whereas anti-inflammatory ones were nearly undetectable. L-PRP administration did not modulate significant changes of cytokine concentrations either in synovial fluid or plasma, whatever the time points analyzed. No different trend was observed between L-PRP and HA administration in terms of pro- and anti-inflammatory cytokines, as well as growth factors.

**Conclusions:**

In contrast with the evidence reported by “in vitro” studies, where a cellular pro-inflammatory response appears to be induced by the presence of leukocytes, these results suggest that the presence leukocyte-rich PRP doesn’t induce a relevant in vivo up regulation of pro-inflammatory mediators.

## Introduction

Osteoarthritis (OA) is a common disease that will affect almost half the population at some point in their life with a marked social impact [1,2]. New options are currently proposed to treat earlier stages of joint degeneration [3,4]. Since results offered by current surgical regenerative treatments, are not satisfactory for this kind of patients [5], research efforts have been directed towards the development of minimally invasive strategies to provide a symptomatic improvement by influencing joint homeostasis.

In this landscape, a novel promising injective treatment is Platelet-rich Plasma (PRP), a blood derivative that has a higher platelet concentrate than whole blood. Platelets release, upon activation, a group of biologically active proteins that promote cellular recruitment, growth, and morphogenesis, and modulate the inflammatory response, as well [6]. Evidence to support PRP use has been initially gathered from the role played in tissue healing by several of the platelet derived growth factors, and the rational has been then confirmed by overall positive findings in both in vitro and in the animal model [7,8].

Based on this biological rational and the preclinical evidence, platelet concentrates have been used as minimally invasive injective treatment for cartilage degeneration and OA since almost a decade and recently some randomized controlled trials documented good results both with respect to placebo and viscosupplementation [9].

However, beside overall positive results, at a deeper analysis the published findings are not consistent and sometimes contradictory both in terms of potential and limitations [9].

Among the possible explanations for the contradictory findings, differences in cellular component among products have been accounted as one of the key factors. In particular, the presence of leukocytes and their contribution in inducing clinical and biological effects is still a debated issue, since some researchers consider them as a source of cytokines and enzymes that may also be important for the prevention of infections, whereas other authors attribute better results to leukocyte depletion, because of the supposed deleterious effects of proteases and reactive oxygen species released from white cells [7,8]. Some in vitro studies, designed to directly compare PRP formulations with or without leukocytes, underlined their inflammatory contribute suggesting potential noxious effects on the treated joints [10–12], while others showed more complex effects with less conclusive findings both in terms of molecule release and cellular influences on chondrocytes and synoviocytes [13,14]. Unfortunately, the clinical significance of all the preclinical findings is difficult to establish due to the lack of trials focused on this aspect. In fact, at present day only one comparative clinical study has been published with no conclusive results. While leucocyte-rich PRP (L-PRP) presented more adverse events such post injective pain and swelling with respect to leukocyte-poor PRP, no differences were documented in the clinical outcome up to 12 months of follow-up [15].

On one side in vitro studies document a net effect towards an inflammatory activation, given the capacity of this preparation to induce a long-term enhancement of pro-inflammatory and pro-catabolic factors thus supporting the discussed hypothesis that leucocytes in PRP might foster unwanted effects, on the other side a possible modulation of joint homeostasis toward an inflammatory response remains a mere speculation due to the lack of clinical evidence.

Further insights into the mechanisms of action of intra articular PRP and on the possible deleterious effects of leukocyte-rich products would require a direct analysis of the net effects on the joint environment.

Thus, the main aim of this study was to analyze joint changes induced by leukocyte-rich PRP by performing a proteomic characterization of serial synovial fluid samples obtained by subjects treated for knee OA, and to compare the effects on knee homeostasis with those obtained with another commonly used injective treatment (hyaluronic acid—HA). Secondary aim was to evaluate if this local treatment may induce changes in systemic response through the comparative analysis of serial peripheral blood samples harvested by patients randomly treated with either a leukocyte-rich PRP or visco-supplementation.

## Methods

### Ethics Statement

The clinical trial was approved by the Rizzoli Orthopedic Institute Ethic Committee and patients signed an informed consent before being enrolled. The approval was granted on 21^st^ January 2009 (protocol number: 2160; S1 File). Originally, the trial did not include the biologic evaluation, so the Authors proposed an “Addendum to the Study Protocol”, which was approved by the Ethical Committee on 19^th^ January 2010 (protocol number 10358; S2 File), thus authorizing all the biologic evaluations and outcome measures described in the present manuscript. The Study Protocol was retrospectively registered on Clinicaltrial.gov (Clinicaltrials.gov Identifier: NCT01670578) due to the fact that, at the moment of starting the trial (2009) the Authors were not aware of the strict necessity of registering a randomized controlled trial on an online-based registry. The trial and the related outcome measures have been then registered to guarantee public access to the study protocol.

### Study design and patient selection

In this study we analyzed 36 out of 192 patients from a previously published double-blinded randomized trial [16]. Briefly, patients affected by a monolateral lesion with a history of chronic (4 months, at least) pain or swelling of the knee and imaging findings of degenerative changes in the joint (Kellgren Lawrence ≤ 3), were included in the study. The treatment (L-PRP or Hyaluronic Acid-HA) was assigned by means of a double-blind randomised procedure. All the patients underwent blood harvesting to obtain autologous PRP, to ensure the blinding of the patients, but L-PRP was injected only in half of them according to the randomization list. One week after blood harvesting, patients were treated with 3 weekly intra articular injection of L-PRP or HA. Details of patients’ selection, study design, randomization and intervention and sample size calculation, as well as Consort 2010 Flow diagram are widely reported by Filardo et al [16]. All 192 patients underwent, at the moment of each intraarticular injection, an attempt for synovial fluid collection. In 36 cases, according to the presence of swelling and/or patient tolerance to the aspiration procedure, synovial fluid was obtained before each injection. Furthermore, a peripheral blood sample was obtained from each subject before the treatment, after each injection, and at 2, 6, and 12 months’ follow up (from February 2010 to February 2013).

### Synovial fluid and blood collection

In concomitance with L-PRP or HA administration, just before the injection, a small sample (1 ml) of synovial fluid was collected through the same needle way aspiration, in 36 patients. In the same time, a peripheral blood sample was collected in a tube with sodium citrate, plasma was obtained by centrifugation and divided in aliquots. A blood sample was also obtained in concomitance with the 2-, 6- and 12- month follow-up.

Both synovial fluid and plasma were cryopreserved al -30°C until used for the soluble factor evaluation.

### Leukocyte-rich plasma (L-PRP) preparation and activation

Venous blood sample (150 ml) was collected in a bag containing 21 ml sodium citrate and centrifuged 15 minutes at 730g. The resulting plasma and buffy-coat, deprived of the majority of the red blood cells, was transferred in a new bag through a closed circuit. Then a second centrifugation at 3800g for 10 minutes was performed, allowing the stratification of platelet poor plasma in the upper part and of L-PRP over the red blood cells [17]. L-PRP was collected and divided in aliquots that underwent the immunological analyses, determination of platelet concentrations and injection. Aliquots for the immunological characterizations and for injections were cryopreserved at -30°C until use.

Before immunological characterizations L-PRP preparations were activated with 10% CaCl_2_ (22.8 mM final concentration) and incubated for 1 hour at 37°C in 5% CO_2_. At the end of the incubation, samples were centrifuged 15 minutes at 2800g at 20°C and the supernatants released from retracted clots were collected and frozen at -80°C until use.

### Determination of platelet and leukocyte numbers in L-PRP

Platelet and leukocyte count were performed by the Coulter LH 750 (Beckman Coulter Inc. Miami, Fl, USA) automated hematology analyzer. Linearity was 5–1000 x 10^3^/μl for platelet count and 0.1–100 x 10^3^/μl for white blood cell count.

In L-PRP, the median platelet number was 1,056x10^3^/μl (interquartile range 827–1,7041x10^3^/μl) and the median leukocyte count was 8.8 x10^3/^μl (interquartile range 6.8–17.3x10^3^/μl).

### Soluble factor evaluation

L-PRP, plasma and synovial fluid obtained by each subject were tested for the presence of selected pro- and anti-inflammatory cytokines (IL-1beta, IL-6, 8, 17 and IL-4, 10, 13, respectively), as well as selected growth factors (FGF-b, HGF, PDGF-AB/BB). Before each assay, all samples were centrifuged to remove debris (S3 File). The viscosity of synovial fluid samples was reduced by treatment by hyaluronidase (Type IV-S, Sigma-Aldrich, St. Louis, Missouri, USA) at a concentration of 20U/ml for 30 min at 37°C, followed by centrifugation [18].

Samples, in duplicate, were simultaneously evaluated, using commercially available multiplex bead-based sandwich immunoassay kits (Bio-Rad Laboratories, CA, USA), by means of the Bio-Plex Protein Array System (Bio-Rad Laboratories, CA, USA) as previously described [19, 20]. This system allows simultaneous quantitative analysis of multiple different factors in a single microtiter well. Briefly, distinct sets of fluorescently dyed beads loaded with capture monoclonal antibodies, specific for each cytokine to be tested, were used. The formation of different sandwich immune complexes on distinct bead sets was measured and quantified. Values with a coefficient of variation above the 10% were discarded before the final data analysis.

Data were analyzed by the Bio-Plex Manager software version 6.0 (Bio-Rad Laboratories, CA, USA). Standard levels between 70 and 130% of the expected values were considered to be accurate and were used. In general, at least six standards were accepted and used to establish standard curves following a Five-Parameter Logistic (5-PL) regression model. Sample concentrations were immediately interpolated from the standard curves.

### Statistical analysis

Values are presented as medians, interquartile ranges, means and standard deviations, as appropriate. Differences of cytokine concentrations among times were analyzed using the Friedman-ANOVA test. Differences within groups were analyzed by the Wilcoxon matched pair test; differences between groups were analyzed by the Mann-Whitney U test. In order to obtain 0.8 power of the Wilcoxon signed-rank test to compare the difference between the IL-1b values pre- and post- treatment, with a basal value of 0.22 pg/ml and a standard deviation of 0.09 of the variation (stating that the confidence interval at 95% of the difference between pre- and post- treatment of IL-1b in the synovial fluid should exceed more than 25% (0.055) of the basal value), the minimum number of patients required is 13 patients undergoing PRP treatment, assuming a one sided alpha level of 0.05.

The level of statistical significance was set at p< 0.05. Data were analyzed using the Statistica 6 software (StatSoft. Inc., Tulsa, USA).

## Results

### PRP characterization

The cytokine concentrations obtained from the injected L-PRP are reported in detail in Table 1.

**Table 1:** Cytokines concentrations in L-PRP.

IL-1b	IL-6	IL-8	IL-17	IL-4	IL-10	IL-13
0.79 [0.47–1.92]	1.61 [0.27–3.18]	14.36 [7.42–28.25]	0.095 [0.00–2.06]	0.00 [0.00–0.00]	0.00 [0.00–5.70]	0.34 [0.00–1,10]

Data are expressed as median pg/ml and [interquartile ranges] (n.19 patients for L-PRP treatment group)

The cytokines released from L-PRP showed physiological variations in their concentrations: the highest observed one was IL-8 followed by IL-6, IL-1b and IL-13, whereas IL-17, IL-10 and IL-4 were almost undetectable.

### Baseline analysis

Patient groups were homogeneous for age, sex, and BMI, as shown in detail in Table 2.

**Table 2:** Characteristics of patients included in the two treatment groups.

	HA	L-PRP	p value
Patient number	17	19	
Age (years)^#^	63 ± 11	58 ± 9	0.071*
Sex	8M, 9F	9M, 10F	0.985**
BMI^#^	27.9 ± 3.4	26.9 ± 3.4	0.512*

^#^Data are expressed as mean ± standard deviation.

*Mann-Whitney U test.

**Chi Square Test.

Both plasma and synovial fluid underwent full analysis of pro- (IL-1b, IL-6, IL-8, IL-17A) and anti-inflammatory (IL-4, IL- 10, IL-13) cytokines.

Baseline characterization of L-PRP and HA treated patients showed similar local (synovial fluid) and systemic (plasma) conditions (Table 3).

**Table 3:** Baseline plasma and synovial fluid cytokines concentrations.

Cytokines (pg/ml)	Treatment	Plasma	Synovial fluid	Plasma vs synovial fluid (p value)
**IL-1b**	**HA**	0.12 [0.00–0.29]	0.17 [0.02–0.35]	p = 1.0000
	**L-PRP**	0.28 [0.20–0.40]	0.20 [0.15–0.34]	p = 0.2858
**IL-6**	**HA**	0.42 [0.00–0.86]	45.59 [11,47–507.26]	*p = 0*.*0005*
	**L-PRP**	0.08 [0.00–0.86]	45.59 [0.87–140.75]	*p<0*.*0001*
**IL-8**	**HA**	0.00 [0.00–1.36]	14.80 [4.73–89.44]	*p = 0*.*0001*
	**L-PRP**	1.18 [0.00–2.34]	6.77 [2.76–19.08]	*p = 0*.*0003*
**IL-17**	**HA**	0.00 [0.00–0.55]	0.00 [0.00–0.12]	Not relevant
	**L-PRP**	0.00 [0.00–0.15]	0.00 [0.00–0.14]	Not relevant
**IL-4**	**HA**	0.00 [0.00–0.00]	0.00 [0.00–0.00]	Not relevant
	**L-PRP**	0.00 [0.00–4.33]	0.00 [0.00–0.00]	Not relevant
**IL-10**	**HA**	0.00 [0.00–1.71]	0.00 [0.00–0.00]	Not relevant
	**L-PRP**	0.00 [0.00–0.73]	0.00 [0.00–0.00]	Not relevant
**IL-13**	**HA**	0.00 [0.00–0.73]	7.26 [1.55–26.85]	p = 0.0591
	**L-PRP**	0.19 [0.00–0.85]	5.32 [2.04–11.69]	*p = 0*.*0231*

Data are expressed as median pg/ml and [interquartile ranges]. Comparison between plasma and synovial fluid was performed by Wilcoxon-matched pair test. (n.17 and n. 19 patients for HA or L-PRP treatment group, respectively)

At baseline, IL-1b concentration was similarly low both in plasma and synovial fluid, IL-6, IL-8 and IL-13 were mostly detectable in synovial fluid, whereas IL-17A, IL-4 and IL-10 concentrations were almost undetectable (Table 3).

IL-1b and IL-8 appeared to be significantly more concentrated in the PRP preparations (Table 1) than in plasma samples (Table 3) (Wilcoxon-matched pair test: p<0.0005 for IL-1b; p<0.0002 for IL-8) and synovial fluid (Table 3) (Wilcoxon-matched pair test: p<0.005 for IL-1b; p<0.05 for IL-8). IL-6 was more concentrated in L-PRP (Table 1) than in plasma samples (Table 3) (Wilcoxon-matched pair test: p<0.002), but less than in synovial fluid (Table 3) (Wilcoxon-matched pair test: p<0.005), whereas IL-13 concentration was lower in L-PRP than in synovial fluid (Table 3) (Wilcoxon-matched pair test: p<0.01).

The analysis of plasma and paired synovial fluid cytokine levels (Table 3) showed a higher local concentration of IL-6, IL-8, and IL-13 (nearly significant in HA group) compared to systemic distribution, whereas IL-1b concentration was similar in plasma and synovial fluid (Table 3).

### Longitudinal and cross-sectional analysis

**Fig 1:**
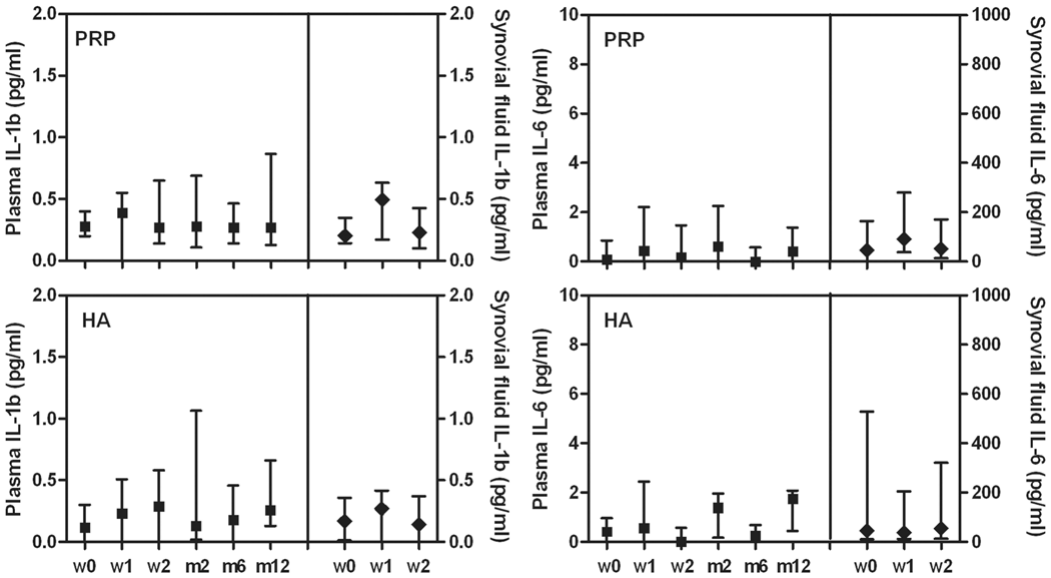
Plasma and synovial fluid concentration of IL-1b and IL-6 in patients treated with L-PRP or HA. Data are expressed as medians and interquartile ranges; comparisons among time points, as determined by Friedman-ANOVA test, and between L-PRP and HA treatments, as determined by the Mann-Whitney U test, are not significant. w = week, m = month

**Fig 2:**
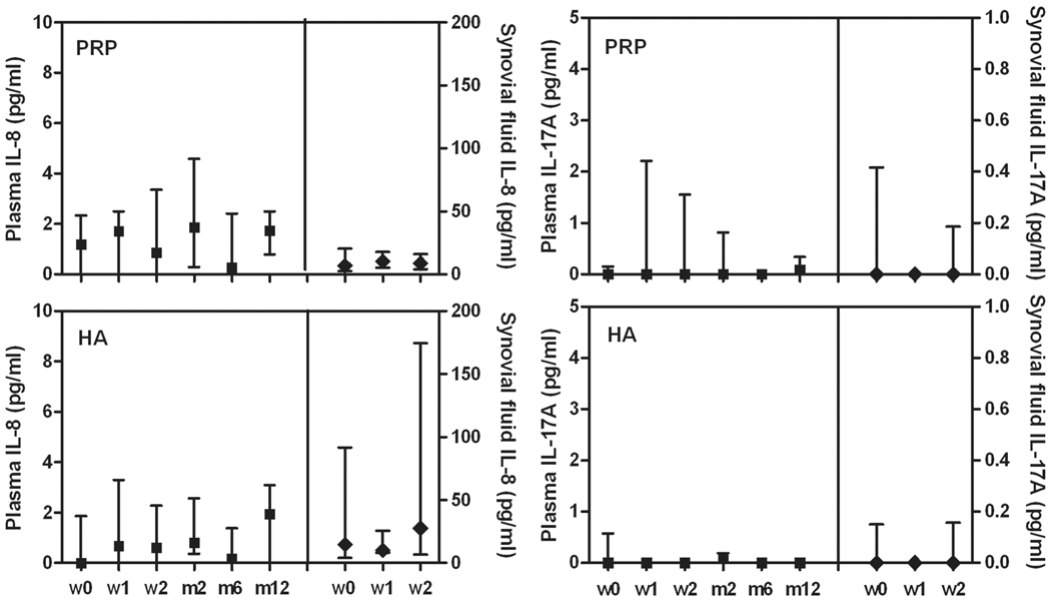
Plasma and synovial fluid concentration of IL-8 and IL-17 in patients treated with L-PRP or HA. Data are expressed as medians and interquartile ranges; comparisons among time points, as determined by Friedman-ANOVA test, and between L-PRP and HA treatments, as determined by the Mann-Whitney U test, are not significant, excluded the comparison between IL-8 concentration in the synovial fluids at w2, p<0.05. w = week, m = month

**Fig 3:**
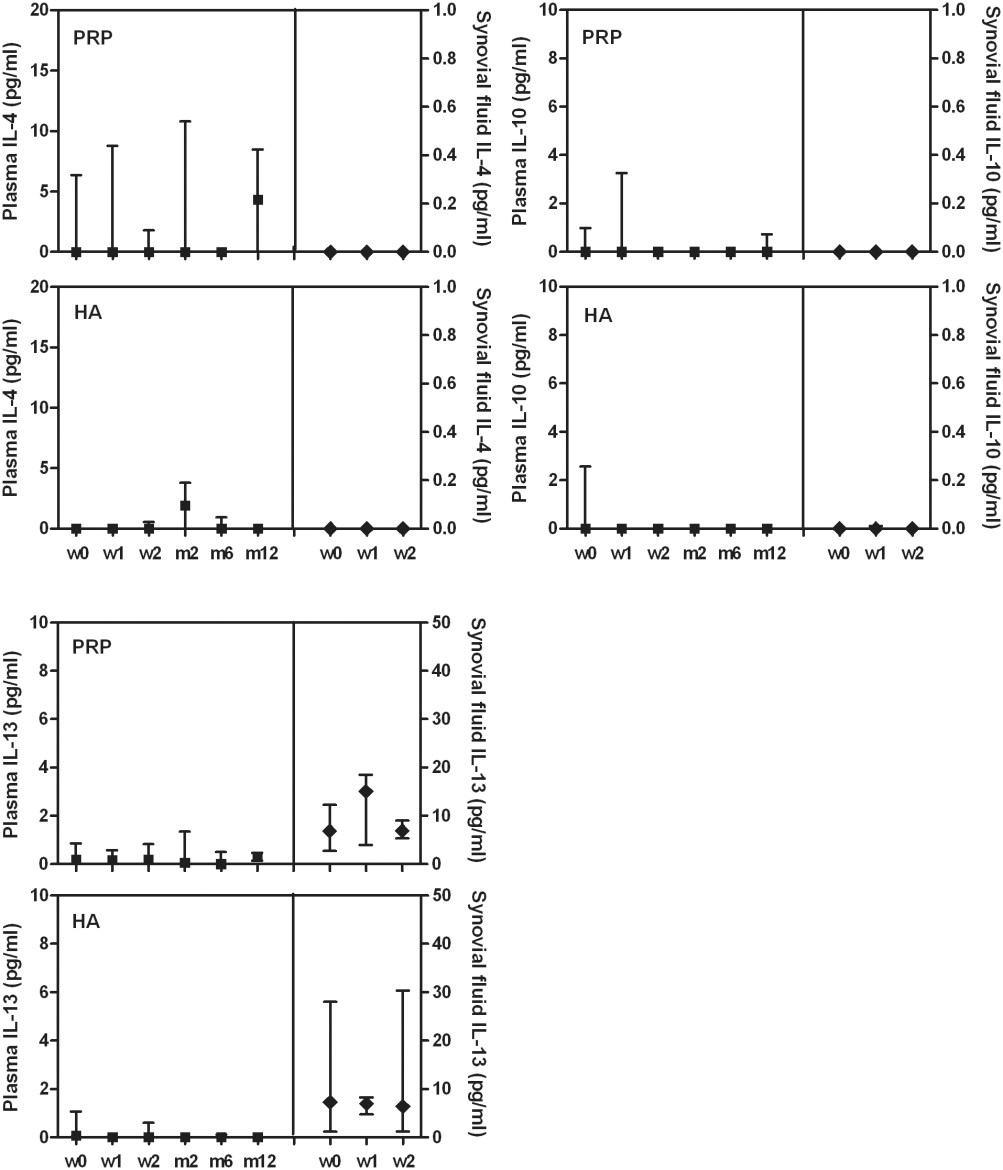
Plasma and synovial fluid concentration of IL-4, IL-10 and IL-13 in patients treated with L-PRP or HA. Data are expressed as medians and interquartile ranges; comparisons among time points, as determined by Friedman-ANOVA test, and between L-PRP and HA treatments, as determined by the Mann-Whitney U test, are not significant. w = week, m = month

Tested plasma and synovial fluid cytokine levels showed no differences among time points of the follow-up period whatever the treatment group (Figs 1–3).

Furthermore no differences were observed by the comparison of L-PRP and HA treatments at any time point (Figs 1–3), excluding IL-8 (Fig 2) that presented a significant higher level in the synovial fluid of HA treated patients at week 2 compared to L-PRP treated patients (Mann-Whitney U test: p<0.05).

### Local growth factors analysis

The growth factor concentrations observed in L-PRP are reported in detail in Table 4.

**Table 4:** Growth Factors levels in L-PRP.

FGF-b	HGF	PDGF-AB/BB
0 [0–25.23]	399.30 [161.27–595.94]	11445.51 [5201.99–35683.88]

Data are expressed as median (pg/ml) and [interquartile ranges]; no. 19 patients for L-PRP treatment group.

**Fig 4:**
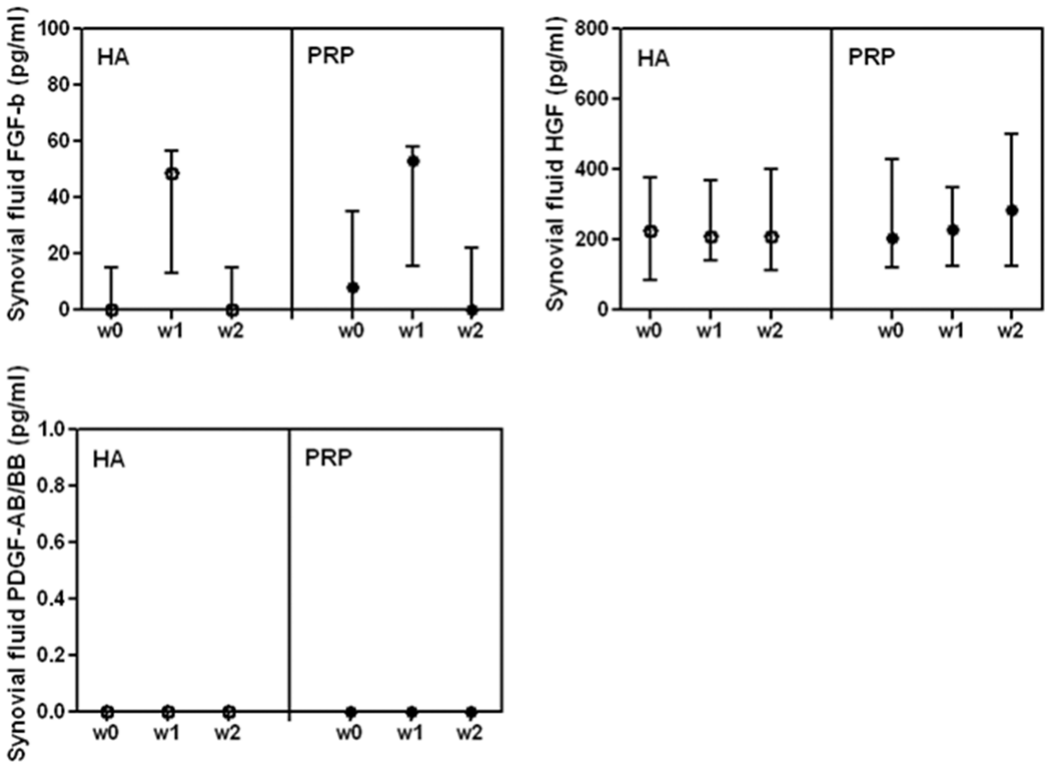
Synovial fluid concentration of FGF-b, HGF and PDGF-AB/BB in patients treated with L-PRP or HA. Data are expressed as medians and interquartile ranges; comparisons among time points, as determined by Friedman-ANOVA test, and between L-PRP and HA treatments, as determined by the Mann-Whitney U test, are not significant. w = week

In L-PRP, the concentration of FGF-b and HGF was similar to the one detected in the synovial fluid, whereas PDGF-AB/BB, not detectable in the synovial fluid (Fig 4), was present in high concentration in L-PRP (Table 4).

L-PRP and HA treated patients showed similar local (synovial fluid) conditions at baseline (Fig 4) and at any following observation (Fig 4). Furthermore FGF-b, HGF and PDGF-AB/BB concentrations in synovial fluids were stable among time points of the follow-up period whatever the treatment group.

## Discussion

Despite the extensive therapeutic application of PRP, the evidence on its efficacy is still controversial in clinical and preclinical studies. One of the main critical issue concerns the different preparation approaches that result in remarkable differences in platelet concentrations and proportions of cellular populations. In particular, the presence of leukocytes and their contribution to the induction of clinical and biological effects is still an open and widely debated issue [21].

Given the complexity of OA, currently considered as a whole joint disease [22], and the relevant cross-talking that occurs between the different joint compartments, the understanding of the biological bases underlying PRP clinical efficacy remains one of main challenges in this field.

A complex interplay of biomechanical, metabolic, and biochemical factors has been thought to contribute to progressive OA cartilage damage by inducing and maintaining an unbalance between anabolic and pro-inflammatory/catabolic activities [23]. In this study the local and systemic modification of a panel of molecules implicated in the physiopathology of the joint environment, including inflammatory (IL-1 beta, IL-8/CXCL8, IL-6, IL-17) and anti-inflammatory (IL-4, IL-10, IL-13) cytokines were explored in patients treated for knee OA by leukocyte-rich PRP and compared with another commonly used injective treatment: HA. Furthermore, among the several different growth factors contained in PRP, we selected three molecules (FGF-b. HGF and PDGF-AB/BB) described as inducers of joint metabolism, chondrocyte proliferation and stem cell migration to damaged osteo-articular tissues. In addition, as concerning PDGF, it is one of the most produced factors by activated platelets and could be considered as a crucial molecule of the anabolic pathway in OA.

The pattern of selected cytokines and growth factors was firstly characterized in L-PRP in order to define the level of each cytokine in the injected preparation.

This analysis demonstrated that in L-PRP (activated with CaCl_2_ and incubated for one hour), almost all evaluated cytokines (excluded IL-4) were bio-available, even if most of them were barely detectable. Time related releases, obtained in vitro from L-PRP preparations, demonstrated peculiar kinetics for various cytokines with increasing concentrations up to seven days [13]. In particular this feature has been reported for IL-1b, detected at low levels in platelets within one hour from thrombin activation and increasing up to 18 hours [24]. This evidence is due to the beginning of IL-1b synthesis only following platelet activation and it is known as signal-dependent translation. We cannot exclude that the activation procedure used in this study can account for the differences observed compared to other authors, since the release of soluble factors from CaCl_2_ activated PRP clot is slower than the one obtained after thrombin. Furthermore it was recently demonstrated that the PRP clot can retain a discrete amount of soluble factors up to 8 days [25].

Since IL-1b is mainly produced by activated leukocytes, the low concentration of this cytokine in L-PRP releasate suggests a negligible contribute of these cells to the baseline systemic inflammatory conditions.

The observation of higher IL-6, IL-8, and IL-13 concentrations in synovial fluid than in plasma confirms a local independent production of these cytokines in OA joints [26, 27]. The differences between the local and systemic compartments are not univocally described and they appear to be variable depending on the disease severity, joint localization, level of local inflammation and sensitivity of the detection method.

Over the longitudinal observation period, no time related modifications of plasma and synovial fluid cytokine concentrations were observed in both groups, suggesting that L-PRP compared to HA administration does not sustain a significant boost of pro- or anti-inflammatory selected cytokines up to seven days after each injection.

In particular, as concerning L-PRP, these results suggest that the presence of leukocytes did not induce a relevant in vivo up regulation of pro-inflammatory mediators, in contrast with the evidence reported by “in vitro” studies, in which the cellular pro-inflammatory response was reported [13, 28]. However, we cannot exclude a short time response, as occur in animal models in which an increase of IL-6 was observed within six hours after PRP injection [29].

Similarly to the pro-inflammatory component also the analysis of the group of anti-inflammatory cytokines (IL-4, IL-10 and IL-13) showed negligible concentrations in the three fluids analyzed, excluding a modulation of the local and systemic response.

Nevertheless, since our results showed no up-regulation of inflammatory mediators at seven days following PRP injection, the potential short-time detrimental effects may be ascribed to only temporary, mild and self-limiting inflammatory effects of leukocytes.

This hypothesis is supported by the results obtained from cross-sectional analysis: in fact, a slight down-modulation of IL-8 was documented in synovial fluid after L-PRP injection compared to HA, despite the inflammatory factor content of injected L-PRP. In addition, concerning the other soluble molecules investigated in this study, no differences between the two treatments were observed in plasma and synovial fluid at each time point.

As concerning FGF-b, HGF and PDGF-AB/BB growth factors analysis, we did not observe a modification of their concentration in the synovial fluid after each consecutive injection, either when present in L-PRP in physiological (as FGF-b and HGF) or in supra-physiological (as PDGF-AB/BB) concentrations. These observations may suggest an in vivo uptake through binding to specific receptors. Since these factors display anti-apoptotic properties for chondrocytes, decrease inflammatory response and increase cartilage ECM synthesis, this potential mechanism could be advantageous for improving the anabolic effects in joints affected by an osteoarthritic process. This hypothesis is supported by studies in vitro demonstrating that the addition of L-PRP to OA chondrocyte and synoviocyte cultures induced a modulation of gene expression for a panel of soluble molecules [13, 14] and also by different anabolism/catabolism patterns displayed by cells from different joint compartments [30].

Nevertheless, we cannot exclude that intra-articular degradative processes could make these molecules not bio-available, also considering the time course of these molecules, which present a stable or increasing release from activated L-PRP up to seven days in vitro (personal un-published observations), and should be therefore detectable in the synovial fluid analysis.

Taking into account the pattern of investigated molecules and their role in joint homeostasis, the overall results that emerge from this study suggest that the net effect of L-PRP does not prompt an inflammatory activation in OA joint compartment at the observed time points, while it supplies growth factors readily available for articular cell compartments.

The widely debated hypothesis that leukocytes in PRP might foster unwanted effects is sustained by other authors essentially in “*in vitro*” studies [28, 31], but this assertion remains a mere speculation. As underlined by our preliminary results, “*in vitro*” studies cannot completely mirror the complexity of joint environment, indeed multiple different cellular populations are involved in the pathophysiology of articular compartment and their cross-talk with a peculiar network of soluble factors actively and collectively operate modulating the joint response [32, 33], which in this case didn’t result in an inflammatory homeostatic leukocyte-induced change after L-PRP one week post-injection.

Some weakness of the current study design might limit the conclusion that can be drown from these data. Due to obvious ethical reasons related to synovial fluid collection, the results reported in this study are limited to a fixed post injection time, so there is lacking evidence on short time-course modifications of cytokine concentrations at local level. However, the samples were collected after one week post-injection, a reasonable time to allow the detection of significant inflammatory changes induced in the treated joint. Despite the high sensitivity of the detection method, the very low levels of some cytokines may have limited the statistical power of the analysis. Finally, another limitation is the relatively small sample size of patients with synovial fluid samples, who were a sub-population of the treated patients and thus possibly not fully representing the general response to PRP treatment. Nonetheless, since patients with effusion (and therefore synovial fluid available for collection and analysis) in the post-injective period may represent an OA population less responsive to treatment, the absence of injection-related inflammatory cytokine up-regulation even in the less successful patients further corroborates the lack of a significant inflammatory effect one week after the administration of L-PRP.

## Conclusions

Intra-articular injection of L-PRP does not modify systemic and local levels of pro-inflammatory cytokines (IL-1b, IL-6, IL-8, IL-17) in OA patients, within one week after treatment. These results suggest a minimal impact of L-PRP inflammatory/catabolic components in diseased joints together with a negligible modulation of anti-inflammatory/anti-catabolic mediators (IL-4, IL-10, IL-13) that appear to be practically undetectable. Further research efforts are needed to clarify the “in vivo” real role of leukocytes on the bioactivity of L-PRP, since leucocyte-platelet interaction may promote the biosynthesis of other factors that counteract or facilitate the resolution of inflammation. Finally, no significant differences could be detected between L-PRP and HA-induced cytokine modulation in these patient groups, both locally and systemically.

## Supporting Information

S1 File Study Trial Protocol (English Translation) (PDF)

S2 File Addendum to the Study Trial Protocol (English Translation) (PDF)

S3 File Concentration of the evaluated molecules, in both treatment groups (all patients’data). (XLSX)
