# Supplementary material for: Leukocyte-Rich Platelet-Rich Plasma Injections Do Not Up-Modulate Intra-Articular Pro-Inflammatory Cytokines in the Osteoarthritic Knee
Source: PLoS One. 2016 Jun 3;11(6):e0156137. doi: 10.1371/journal.pone.0156137 (PMC4892682; doi:10.1371/journal.pone.0156137)
Supplement: S1 File — (PDF) [file pone.0156137.s001.pdf]

SERVIZIO SANITARIO REGIONALE
EMILIA - ROMAGNA
Istituto Ortopedico Rizzoli di Bologna
Istituto di Ricovero e Cura a Carattere Scientifico

BOLOGNA, 19-01-2009

TRIAL PROTOCOL
“The use of PRP for the treatment of knee degenerative lesions: a RCT”
(ENGLISH TRANSLATION OF THE ORIGINAL PROTOCOL)

Description of the study

Current research is investigating new methods for stimulating repair or replacing damaged cartilage. In particular, the most recent knowledge regarding tissue biology highlights a complex regulation of growth factors (GFs) for the normal tissue structure and the reaction to tissue damage. The influence of GFs on cartilage repair is now widely investigated in vitro and in vivo. Platelet Rich Plasma (PRP) is a simple, low- cost and minimally- invasive method that allows one to obtain a natural concentrate of autologous GFs from the blood, and it is increasingly applied in the clinical practice to treat knee degenerative pathology, such as chondropathy and early OA. The biological rational of PRP is that platelets contain storage pools of GFs, cytokines, chemokines and many other mediators.Although its widespread application, there are no high level studies in the literature to demonstrate the real efficacy of PRP. In fact, at the present moment, to our knowledge there is no published randomized controlled trial comparing PRP with other conservative treatments commonly used for knee OA.

The investigators hypothesized that intra-articular injections of PRP to treat knee degenerative articular cartilage pathology could determine pain relief and recovery of knee function with overall clinical outcome comparable or even better than viscosupplementation, which is a common injective approach applied in this kind of

pathology. To this purpose the investigators designed a double blind randomized controlled trial comparing PRP vs viscosupplementation.

A power analysis has been performed for the primary endpoint of IKDC subjective score improvement at the 12-month follow-up for PRP. From a pilot study, a standard deviation of 15.2 points was found. With an alpha error of 0.05, a beta error of 0.2 and a minimal clinically significant difference of 6.7 points corresponding at 1/3 of the documented mean improvement, the minimum sample size was 83 for each group. Considering a possible drop out of 15%, 96 patients per group are required for total 192 patients. Patients are then assigned to two different treatment groups, according to a randomization list. The first group of treatment consists of three weekly intra-articular injections of autologous PRP obtained with the following procedure: a 150-ml autologous venous blood sample undergoes 2 centrifugations (the first at 1480 rpm for 6 minutes to separate erythrocytes, and a second at 3400 rpm for 15 minutes to concentrate platelets) to produced 20 ml of PRP. This unit of PRP is then divided into 4 small units of 5 ml each. One unit is sent to the laboratory for analysis of platelet concentration and for a quality test, 3 units are stored at -30° C.

The second treatment group consists of patients receiving three weekly injections of hyaluronic acid (Hyalubrix 30 mg/2ml, Fidia Farmaceutici Spa, Italy;Molecular Weight: 1500 kDa).

To guarantee the blinding of the patients, all of them undergo blood harvesting to obtain autologous PRP which will be used only in half of them, according to the aforementioned randomization list. One week after the PRP production, the injective treatment starts, with 3 weekly injections of PRP or HA. At the moment of the injection the syringe is properly covered to prevent the patient from discovering the substance he was receiving. After the injection, patients are sent home with instructions to limit the

use of the leg for at least 24 h and to use cold therapy/ice on the affected area to relieve pain. During this period, the use of non-steroidal medication is forbidden.

Patients are prospectively evaluated basally and at 2, 6, and 12 months of follow-up using IKDC, KOOS (Knee Injury and Osteoarthritis Outcome Score), EQ-VAS (Visual Analogue Scale) for general health status, and Tegner scores. Furthermore at basal evaluation and at every follow-up the ROM (Range of Motion) and the transpatellar circumference of both the index knee and the contralateral one are measured to check if any changes occurred over time. Patient satisfaction and adverse events will be also reported. All the clinical evaluations are performed by a medical staff not involved in the injective procedure, in order to keep the study double blinded. At the end of the study, the nature of the injected substance is revealed to the patients.

SUMMARY OF INCLUSION/EXCLUSION CRITERIA

Inclusion Criteria:

- age betweem 18 and 80;
- patients affected by knee articular degenerative pathology with history of chronic (for at least 4 months) pain or swelling;
- imaging findings of degenerative changes of the joint (Kellgren Lawrence 0 to III at X-ray evaluation).

Exclusion Criteria:

- age > 80 years;
- Kellgren-Lawrence score at X-ray evaluation > 3;
- major axial deviation (varus >5° , valgus > 5°),

- systemic disorders such as diabetes, rheumatoid arthritis, haematological diseases (coagulopathy), severe cardiovascular diseases, infections, immunodepression;
- patients in therapy with anticoagulants or antiaggregants;
- use of NSAIDs in the 5 days before blood donation;
- patients with Hb values < 11 g/dl and platelet values < 150,000/mmc.

STATISTICAL ANALYSIS

Sample size calculation

A power analysis was performed for the primary endpoint of the IKDC subjective score improvement at the 12-month follow-up. From a pilot study, a standard deviation of 15.2 points was found. With an alpha error of 0.05, a beta error of 0.2 and a minimal clinically significant difference of 6.7 points corresponding to 1/3 of the documented mean improvement, the minimum sample size was 83 for each group. Considering a possible drop out of 15% 96 patients per group were required, for a total of 192 patients who were effectively enrolled.

SUMMARY OF INTERVENTIONS

Experimental: PRP Group

Patients (n=96) randomized to this group of treatment will receive 3 blinded knee intra-articular injections of autologous Platelet-Rich Plasma one week apart each other.

Active Comparator: Hyaluronan Group

Patients (n=96) randomized to this group of treatment will receive 3 blinded knee intra-articular injections of hyaluronic acid (Hyalubrix 30 mg/2ml, Fidia Farmaceutici Spa, Italy) one week apart each other.

SERVIZIO SANITARIO REGIONALE
EMILIA - ROMAGNA
Istituto Ortopedico Rizzoli di Bologna
Istituto di Ricovero e Cura a Carattere Scientifico

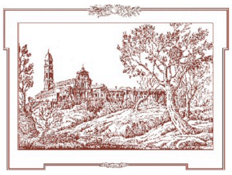

ESSENTIAL REFERENCES

Sánchez M, Azofra J, Anitua E, Andía I, Padilla S, Santisteban J, Mujika I. Plasma rich in growth factors to treat an articular cartilage avulsion: a case report. Med Sci Sports Exerc. 2003 Oct;35(10):1648-52. PubMed ID: 14523300

Everts PA, Knape JT, Weibrich G, Schönberger JP, Hoffmann J, Overdevest EP, Box HA, van Zundert A. Platelet-rich plasma and platelet gel: a review. J Extra Corpor Technol. 2006 Jun;38(2):174-87. Review. PubMed ID: 16921694

Tschon M, Fini M, Giardino R, Filardo G, Dallari D, Torricelli P, Martini L, Giavaresi G, Kon E, Maltarello MC, Nicolini A, Carpi A. Lights and shadows concerning platelet products for musculoskeletal regeneration. Front Biosci (Elite Ed). 2011 Jan 1;3:96-107. Review. PubMed ID: 21196289

Grimaud E, Heymann D, Rédini F. Recent advances in TGF-beta effects on chondrocyte metabolism. Potential therapeutic roles of TGF-beta in cartilage disorders. Cytokine Growth Factor Rev. 2002 Jun;13(3):241-57. Review. PubMed ID: 12486877

Sánchez AR, Sheridan PJ, Kupp LI. Is platelet-rich plasma the perfect enhancement factor? A current review. Int J Oral Maxillofac Implants. 2003 Jan-Feb;18(1):93-103. PubMed ID: 12608674

Hickey DG, Frenkel SR, Di Cesare PE. Clinical applications of growth factors for articular cartilage repair. Am J Orthop (Belle Mead NJ). 2003 Feb;32(2):70-6. Review. PubMed ID: 12602635

Sampson S, Reed M, Silvers H, Meng M, Mandelbaum B. Injection of platelet-rich plasma in patients with primary and secondary knee osteoarthritis: a pilot study. Am J Phys Med Rehabil. 2010 Dec;89(12):961-9. doi: 10.1097/PHM.0b013e3181fc7edf. PubMed ID: 21403592

Wang-Saegusa A, Cugat R, Ares O, Seijas R, Cuscó X, Garcia-Balletbó M. Infiltration of plasma rich in growth factors for osteoarthritis of the knee short-term effects on function and quality of life. Arch Orthop Trauma Surg. 2011 Mar;131(3):311-7. doi: 10.1007/s00402-010-1167-3. Epub 2010 Aug 17. PubMed ID: 20714903

SERVIZIO SANITARIO REGIONALE
EMILIA - ROMAGNA
Istituto Ortopedico Rizzoli di Bologna
Istituto di Ricovero e Cura a Carattere Scientifico

Kon E, Filardo G, Di Martino A, Marcacci M. Platelet-rich plasma (PRP) to treat sports injuries: evidence to support its use. Knee Surg Sports Traumatol Arthrosc. 2011 Apr;19(4):516-27. doi: 10.1007/s00167-010-1306-y. Epub 2010 Nov 17. Review. PubMed ID: 21082164

Kon E, Buda R, Filardo G, Di Martino A, Timoncini A, Cenacchi A, Fornasari PM, Giannini S, Marcacci M. Platelet-rich plasma: intra-articular knee injections produced favorable results on degenerative cartilage lesions. Knee Surg Sports Traumatol Arthrosc. 2010 Apr;18(4):472-9. doi: 10.1007/s00167-009-0940-8. Epub 2009 Oct 17. PubMed ID: 19838676

Filardo G, Kon E, Buda R, Timoncini A, Di Martino A, Cenacchi A, Fornasari PM, Giannini S, Marcacci M. Platelet-rich plasma intra-articular knee injections for the treatment of degenerative cartilage lesions and osteoarthritis. Knee Surg Sports Traumatol Arthrosc. 2011 Apr;19(4):528-35. doi: 10.1007/s00167-010-1238-6. Epub 2010 Aug 26. PubMed ID: 20740273
