# Supplementary material for: Leukocyte-Rich Platelet-Rich Plasma Injections Do Not Up-Modulate Intra-Articular Pro-Inflammatory Cytokines in the Osteoarthritic Knee
Source: PLoS One. 2016 Jun 3;11(6):e0156137. doi: 10.1371/journal.pone.0156137 (PMC4892682; doi:10.1371/journal.pone.0156137)
Supplement: S2 File — (PDF) [file pone.0156137.s002.pdf]

SERVIZIO SANITARIO REGIONALE
EMILIA - ROMAGNA
Istituto Ortopedico Rizzoli di Bologna
Istituto di Ricovero e Cura a Carattere Scientifico

BOLOGNA, 19-01-2010

ADDENDUM TO THE STUDY PROTOCOL TITLED
"The use of PRP for the treatment of knee degenerative lesions: a RCT"

Evaluation of biological effects in synovial fluid and blood

It is of utmost importance to enquire about the biologic mechanisms of action of PRP, both locally, by evaluating synovial fluid, and systemically, by evaluating peripheral blood samples of patients treated. In particular, our aim is to evaluate the local and systemic concentration of pro and anti-inflammatory molecules and also growth factors, that could play a role in modulating cartilage metabolism and, therefore, progression of articular degeneration. Since this double blinded trial entails a randomization of patients, treated either by PRP or hyaluronic acid (HA), it will be possible to examine the biologic effects of intra-articular injections in both treatment groups. To this purpose, each patients will undergo an attempt of synovial fluid aspiration before each therapeutic injection (for a total of three attempts). Furthermore, each patients will undergo a peripheral blood sample (3 ml) during each treatment visit and during each follow-up visit (6 blood samplings in total, considering three evaluations at 2, 6 and 12 months after the injective treatment). Due to invasiveness of synovial fluid aspiration and the related infective risks, no aspiration will be performed during follow-up visits (just blood sampling).
The samples collected (both synovial fluid and blood) will be sent immediately to the Immuno-rheumatology Lab of the Rizzoli Orthopaedic Institute, where they will be stored and analyzed for the purposes of the present study.
The molecules (cytokines and growth factors) to be analyzed will be decided in agreement with the biologists involved in the present trials, based on the evidence from literature and on the funds available. These analyses will provide data about the trend of concentration of the selected molecules over time, both at local level (synovial fluid) and systemically (peripheral blood).

Approved on 24-4-09, protocol number 10358 (see file enclosed in the mail)

Istituto Ortopedico Rizzolii
Via G.C. Pupilli, 1
40136 Bologna, Italy

Istituto Ortopedico Rizzoli
Sede legale Via Di Barbiano, 1/10 - 40136 Bologna
Ospedale Via G. C. Pupilli, 1 - 40136 Bologna
